# Effect of Hyaluronic Acid and Mesenchymal Stem Cells Secretome Combination in Promoting Alveolar Regeneration

**DOI:** 10.3390/ijms24043642

**Published:** 2023-02-11

**Authors:** Francesca Della Sala, Gennaro Longobardo, Gianluca Lista, Francesco Messina, Assunta Borzacchiello

**Affiliations:** 1 Institute of Polymers, Composites and Biomaterials, National Research Council (IPCB-CNR), Viale J.F. Kennedy 54, 80125 Naples, Italy; 2 Department of Chemical, Materials and Production Engineering, University of Naples Federico II, Piazzale V. Tecchio 80, 80125 Naples, Italy; 3 Neonatologia e Terapia Intensiva Neonatale, Ospedale dei Bambini “Vittore Buzzi”, Via Lodovico Castelvetro, 32, 20154 Milan, Italy; 4 Ospedale Evangelico Betania, Via Argine, 604, 80147 Naples, Italy

**Keywords:** Hyaluronic acid, secretome, mesenchymal stem cells, pulmonary differentiation

## Abstract

Pharmacological therapies in lung diseases are nowadays useful in reducing the symptomatology of lung injury. However, they have not yet been translated to effective treatment options able to restore the lung tissue damage. Cell-therapy based on Mesenchymal Stem Cells (MSCs) is an attractive, as well as new therapeutic approach, although some limitations can be ascribed for therapeutic use, such as tumorigenicity and immune rejection. However, MSCs have the capacity to secrete multiple paracrine factors, namely secretome, capable of regulating endothelial and epithelial permeability, decrease inflammation, enhancing tissue repair, and inhibiting bacterial growth. Furthermore, Hyaluronic acid (HA) has been demonstrated to have particularly efficacy in promoting the differentiation of MSCs in Alveolar type II (ATII) cells. In this frame, the combination of HA and secretome to achieve the lung tissue regeneration has been investigated for the first time in this work. Overall results showed how the combination of HA (low and medium molecular weight HA) plus secretome could enhance MSCs differentiation in ATII cells (SPC marker expression of about 5 ng/mL) compared to the only HA or secretome solutions alone (SPC about 3 ng/mL, respectively). Likewise, cell viability and cell rate of migration were reported to be improved for HA and secretome blends, indicating an interesting potentiality of such systems for lung tissue repair. Moreover, an anti-inflammatory profile has been revealed when dealing with HA and secretome mixtures. Therefore, these promising results can allow important advance in the accomplishment of the future therapeutic approach in respiratory diseases, up to date still missing.

## 1. Introduction

By 2030, lung diseases will account for about one in five deaths worldwide, compared to one-sixth of all deaths globally in nowadays, as estimated by the World Health Organization (WHO). Although the etiologies of various lung pathologies are still unclear, their complex pathology is in many cases associated with dysregulation in immune responses, aberrant repair processes of lung tissue, the anomalous remodeling of the extracellular matrix (ECM) and the consequent alteration of respiratory functions [1,2]. In particular, the alveolar type II (ATII) cells represent the key target of the external injury. Indeed, ATII cells are a fundamental gusset in the induction of the repair process to reestablish normal pulmonary function [3]. In effect, ATII cells carry out the essential role of the surfactant production, required to reduce the surface tension between alveolar gases and the hydrated epithelial cell surfaces of the alveoli, allowing the respiration process [4]. Until now, beyond supportive care, there are no effective treatments for lung-injured epithelia. Available pharmacologic approaches are mainly based on anti-inflammatory drugs, such as corticosteroids, and theophylline and bronchodilators. These ones only act to minimize airflow limitation and acute exacerbations and do improve the quality of life of patient but in particular the long-term use of corticosteroids is associated with severe side effects, such as the loss of muscle tissue and the Cushing’s Syndrome [5]. In this scenario, regenerative medicine has the potential to provide innovative new therapies for people with disabling acute and chronic lung diseases. Overall, plenty of preclinical studies have provided convincing evidence of the effectiveness of MSC based therapy in the treatment of many lung disorders. As recently reported, MSCs treatment improved disease-associated parameters in acute respiratory distress syndrome (ARDS) as well as in chronic lung disease of the preterm infants (e.g., bronchopulmonary dysplasia), other chronic obstructive pulmonary diseases, pulmonary hypertension and idiopathic pulmonary fibrosis [6,7,8]. Moreover, it has been reported the ability of MSCs to differentiate in vitro into ATII epithelial cells. Despite the MSCs therapy advantages, it is needed to overcome different limitations of this method, such as tumorigenicity and immune rejection [9]. It is now evident from the literature that MSCs, acting by a paracrine mechanism, can be considered as potent reservoir of biologically active substances collectively known as secretome [10]. These MSCs secretome is made of both soluble proteins, including a broad spectrum of cytokines, chemokines and growth factors and extracellular vesicles (EVs) of micro- and nano-size. Once released, secretome can activate endogenous stem cells and progenitor cells, suppress apoptosis, regulate the inflammatory response, stimulate the remodeling of the extracellular matrix and angiogenesis, reduce fibrosis and mediate the chemoattraction [11]. The employment of secretome in therapy has several advantages compared to MSCs, such as the lack of the potential endogenous tumor formation since it cannot self-replicate, the low immunogenicity, the low emboli formation when intravenously injected, ease of manipulation and storing resulting in a ready to use product suitable for emergency interventions [12]. Therefore, MSC secretome emerges as a promising cell-free therapeutic strategy for the treatment of acute and chronic lung diseases, as it displays the same regenerative properties of parental MSCs. In pulmonary medicine, Hyaluronic acid (HA), a non-sulfated glycosaminoglycan, consisting of repeating polymeric disaccharides, D-glucuronic acid and N-acetyl-D-glucosamine [13], is already widely recognized for its symptom-relieving properties. Moreover, the HA is critical for maintaining the integrity of the ECM, since it is a pivotal component, and homeostasis in the lung parenchyma, where purportedly plays the main role in water regulation in the alveolar interstitium and may play a role in alveolar surface structure stabilizing the surfactant proteins [14]. In a recent study, we have demonstrated that HA, at low and medium molecular weight, can greatly increase the expression of lung surfactant protein, indicating the ability of HA to promote the MSC differentiation in ATII cells [15]. Starting from these promising assumptions, the aim of this work was to investigate, for the first time, the synergistic effect of HA and MSC secretome in promoting lung regeneration. To this aim, first, since in the biomedical field, HA applications are related to their physical-chemical properties, such as molecular weight (MW) [16], HA at 200 (LMW), 500 (MMW), and 1435 (HMW) kDa combined with MSCs secretome has been used to implement the medium to promote the alveolar regeneration. Cell viability and differentiation of umbilical cord mesenchymal stem cells (hUCMSCs) into ATII cells was evaluated by immunohistochemistry analysis assessing the expression of surfactant protein C (SP−C), specific marker of pulmonary differentiation, together with the other pulmonary surfactant proteins A, B, and D, (SP-A, B, and D). To further understanding of the tissue regeneration process, cell migration was investigated in response of biomaterials. Finally, in order to study the inflammatory response of the HA and secretome, Interleukin- 10 and 6 have been evaluated.

## 2. Results

### 2.1. Immunofluorescence Anti-SPC and Anti-CD73

**Figure 1:**
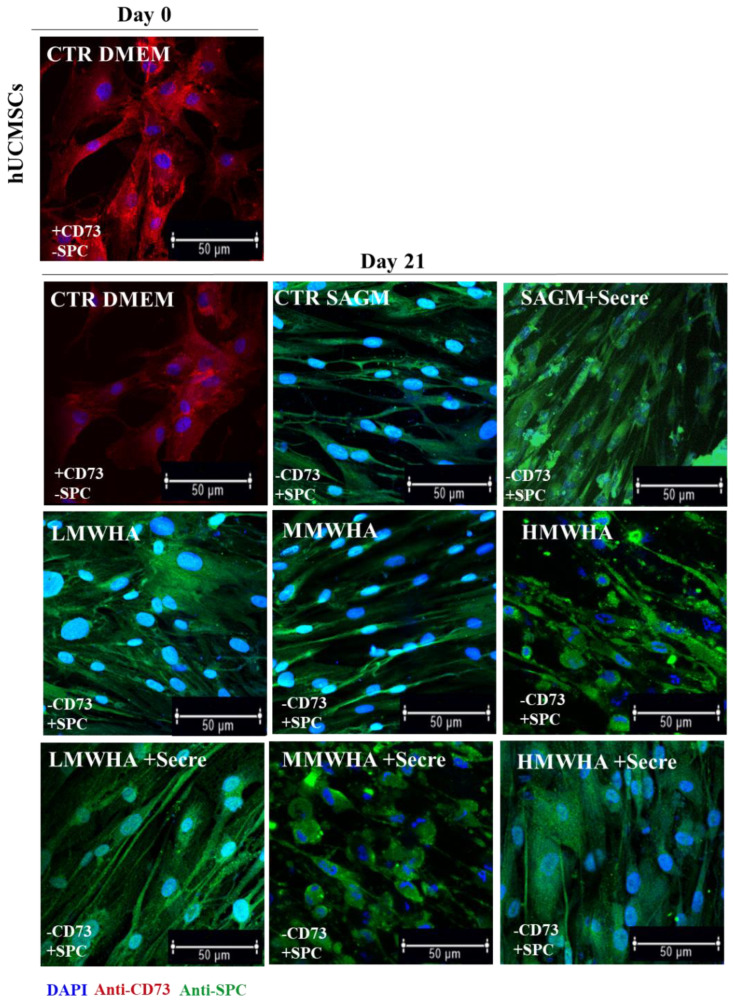
Representative confocal imagines of Immunoreactive SPC cells and CD73 stemness marker expression after the incubation of HA and HA plus secretome solution implementing SAGM at day 0 and after 21 days of incubation. Nuclei were stained with blue DAPI. At Day 0, positive expression of CD73 (red) were detected, indicating the presence of undifferentiated stem cells. Positive expression of SPC (green) in the cytoplasm were assessed after 21 days of exposure with implemented cell-media, indicating the qualitative expression of differentiated ATII cells compared with the untreated control (CTR DMEM). Scale bar: 50 µm.

In order to evaluate qualitatively the differentiation of hUCMSCs into ATII cells following exposure to HA at different MW, to Secretome solutions, and to HA at different MW plus secretome, respectively implementing SAGM, the cells were stained with a specific pulmonary differentiation marker (SPC antibody) and against a stemness marker (CD73 antibody) as a control [17] (Figure 1). At day 0 and similarly after 21 days, only positive expression of CD73 (red) was detected in cells maintained with DMEM alone (as control), indicating the presence of undifferentiated cells. The qualitative expression of SPC (green) has been demonstrated after 21 days of the exposure with LMWHA, MMWHA and HMWHA solutions and for the same solutions implemented with the secretome compared with the cell controls (maintained in only DMEM) clearly negative.

### 2.2. Surfactant Proteins Expression

**Figure 2:**
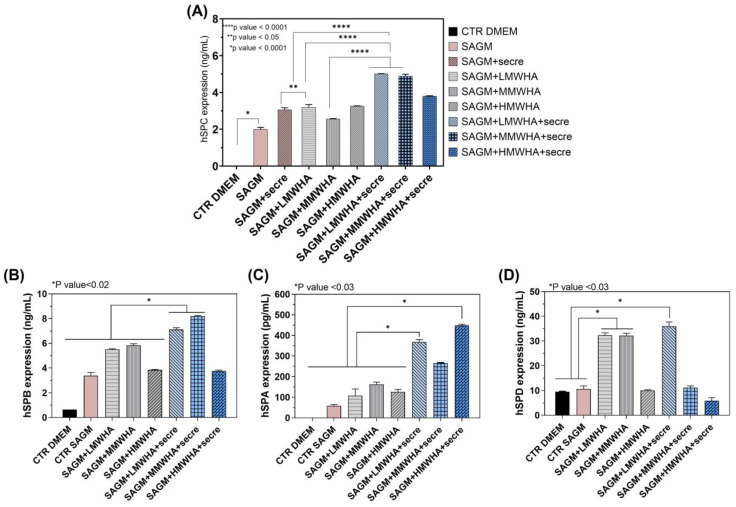
Quantitative expression of SPC (**A**), SP-B (**B**) and SPA (**C**) and SPD (**D**) performed by Elisa kit test, after the incorporation of secretome in combination with HA in SAGM.

The quantitative expression of SPC (Figure 2A) has been performed after the exposure to the HA and secretome solubilised in the SAGM. It has been possible to observe that both HA at different MW and HA at different MW with secretome implementation, attained an increase in expression compared not only to SAGM alone and DMEM controls, but also when compared to the secretome control (SAGM + Secre). Remarkably, the addition of secretome in HA solutions has amplified the amount of the expression obtained by the use of HA biomaterials alone. In particular, the estimated increment was twice times higher (about 5 ng/mL) than the use of SAGM alone (about 2 ng/mL) and once time higher compared to the use of HA solution (about 3 ng/mL). In particular, a better effect of the combination between secretome and HA occurred in combination with the LMWHA and MMWHA, for which a nearly-additive effect in SPC expression can be found with respect to single components (i.e., SAGM + Secre, SAGM + LMWHA and SAGM + MMWHA). As a matter of fact, a high statistical significance of LMWHA + Secre and MMWHA + Secre samples was found when compared to controls and only HA solutions. In addition, as shown in the Figure 2B–D, the other proteins of surfactant SP-B, A and C were expressed in presence of the secretome in the HA solution, and in this case significant differences have been evaluated between the HA solution alone and the addition of secretome to the HA/SAGM media solution. In particular, it can been seen from the Figure 2 that the incorporation of secretome with HA in SAGM media appear always beneficial on the expression.

### 2.3. Cell Viability

**Figure 3:**
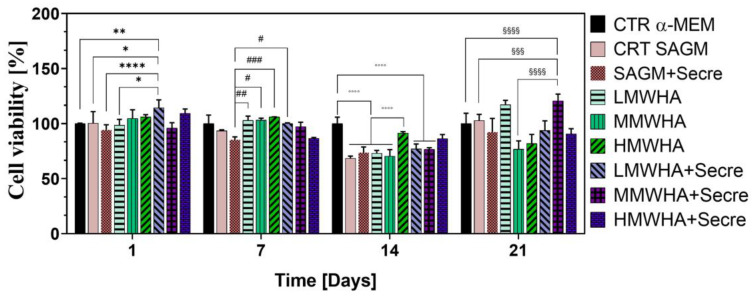
Cell viability performed by Alamar blue test. Viability percentage as function of time at 1, 7, 14 and 21 days of cell culture during the differentiation test. For each evaluated day of culture, all samples’ viabilities are referred to CTR α-MEM as control at that specific day. * *p* value < 0.05, ** *p* value < 0.01, **** *p* value < 0.0001; # *p* value < 0.05, ## *p* value < 0.01, ### *p* value < 0.001; °°°° *p* value < 0.0001; §§§ *p* value < 0.001, §§§§ *p* value < 0.0001.

To investigate the feasibility of using these biomaterials in pulmonary tissue engineering applications the biocompatibility expressed as cell viability, in long-term culture after 21 days of incubation (differentiation time) has been assessed. As shown in Figure 3, overall, the cell viability has been resulted to be good for all sample analyzed. In particular, the viability of cells, compared to the DMEM and SAGM controls, resulted to be greater, over 100%, for the samples with LMWHA and about 120% for MMWHA + Secre, indicating an improvement given by the combination of HA and secretome. A statistical significance can be reported, as a matter of fact, for LMWHA + Secre with respect to all controls (α-MEM, SAGM and SAGM + Secre). After 7 days of incubation secretome-containing samples show a decrement of viability, probably associated with the early establishment of differentiation pathways, it has been widely reported that with the establishment of cellular differentiation signals an inhibition of cellular proliferation occurs, with the establishment of a state of cellular quiescence [18]. After 14 and 21 days of incubation, a decrease in the percentage of viability can be strongly assessed, as both HA and HA secretome samples show a comparable viability, significantly lower than the α-MEM control. Eventually, after 21 days of incubation a decrease in viability percentage can be detected for almost every sample containing secretome or HA plus secretome, with the only exception of MMWHA + Secre, which showed comparable viability with respect to LMWHA sample of about 115%.

### 2.4. Cell Migration Study

**Figure 4:**
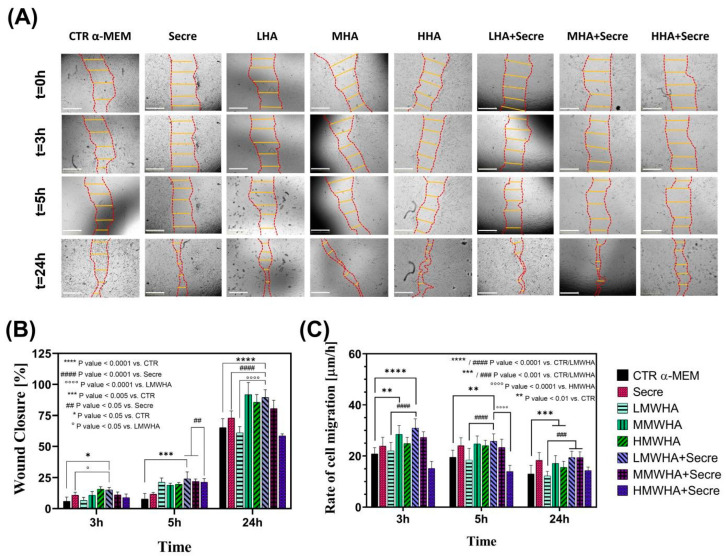
Cell migration study results. (**A**) Bright field microscopy representative images show migration of hUCMSCs cells after scratching at time 0 and after 3, 5 and 24 h of incubation with control medium and solution of HA and HA + Secre. The Regions of Interest (ROI) for wound closure analysis are highlighted with dashed red lines and the solid yellow lines are representative of the ones used to calculate the rate of cell migration. Scale bar: 500 μm. A qualitative reduction in wound area can be detected. (**B**) Wound closure (%) at different times of incubation: a progressive increase can be noticed for all samples. (**C**) Rate of cell migration (μm/h) at different times of incubation. All results are presented as mean ± standard deviation. The data are representatives of eightfold.

Cell migration is a complex dynamic process, which plays an important role in tissue regeneration and repairing. A tissue regenerative biomaterial substrate should provide the basic supporting for the growth of cells and their recruitment and migration, since cell directional migration to the wound site is a crucial prerequisite in tissue regeneration [19]. Bright field microscopy images show migration of hUCMSCs cells after scratching at time 0 and after 3, 5 and 24 h of incubation with the control and formulations. A greater reduction in the wound area can be qualitatively observed following contact with the formulations (Figure 4A). A quantitative analysis indicated the percentage of wound closure at various times (Figure 4B). The data showed that the combination of HA and secretome, in particular for low and medium MW, is more effective in promoting cell migration and therefore wound closure. The results displayed a statistic significance (*p* < 0.05) observed at 5 h of incubation for the LMWHA + Secre solution compared to Secretome sample. The effects on wound closure at 24 h has shown that LMWHA + Secre and MMWHA+Secre solutions is highly significant (*p* < 0.0001) compared to CTR, LMWHA and Secre. Congruent results were found evaluating the rate of cell migration (R_M_) with respect to hours of incubation, observing a general decreasing trend in R_M_. From Figure 4C it can be noticed that LMWHA + secre solution, after 3 h of incubation, shows a high level of significance (*p* < 0.0001) compared to both CTR and LMWHA formulation without secretome. Same considerations can be drawn after 5 h incubation, observing a small reduction in significance between LMWHA + Secre solution and CTR (*p* < 0.01). Eventually, after 24 h Secretome-enriched LMWHA and MMWHA maintained a rate of migration higher than the other samples, with an high significance with respect to control and the LMWHA solution without secretome.

### 2.5. In Vitro Inflammatory Response

**Figure 5:**
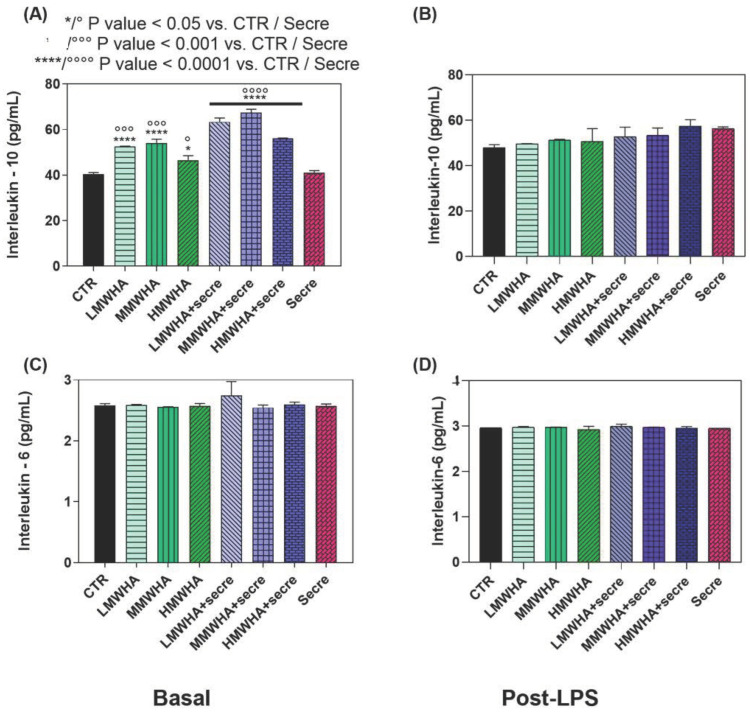
The results of in vitro inflammatory response. Effects of HA and Secretome on basal inflammatory response (**A**,**C**) and after stress through LPS exposure (**B**,**D**). In particular, IL-10 (**A**,**B**) and IL-6 (**C**,**D**) expression on hUCMSCs cells are reported. The results are mean ± of three experiments. * *p* < 0.001 vs. Control.

The regenerative effect of HA and secretome combined has been also analyzed as the capability to inhibit inflammatory reactions in basal and post-insult conditions. To this end, the immune response as inflammatory (IL-6) and anti-inflammatory (IL-10) cytokine expression was estimated before and after exposure to LPS, a well-known endotoxin deriving from the wall of Gram-negative bacteria and widely used as a flogistic stimulus for in vitro studies (Figure 5). As a matter of fact, our results indicated that HA and secretome together do not show significant effect on the expression of IL-6 levels (pro-inflammatory cytokine) and contemporarily significantly increasing of IL-10 levels (anti-inflammatory cytokine) has been observed. It has been observed that the HA solutions alone displayed a good effect on basal immune response of IL-10, in particular for the LMWHA and MMWHA solutions (both about 50 pg/mL). However, the combination with secretome significantly increased such effect compared to untreated controls. These effects on interleukin levels suggest a preventive anti-inflammatory profile of HA + Secre. The highest IL-10 levels were induced by LMWHA + Secre (about 65 pg/mlL) and MMWHA + Secre (about 78 pg/mL) compared the untreated control (40 pg/mL), showing the highest significance (*p* < 0.0001) compared to CTR.

## 3. Discussion

Since one of the key factors in the repair or regenerative process is the ability of local MSCs to proliferate and differentiate to replace damaged cells or tissues, primary, the synergic effect of the HA and secretome to improve the differentiation ability of hUCMSCs into ATII cells has been assessed. Qualitatively, the immunofluorescence shown (Figure 1) ATII positive SPC cells, after 21 days of incubation with all the blends of HA at different MW and for the HA plus Secretome, compared to the untreated controls maintained with only DMEM and expressing the marker of stemness CD 73. Thus the SPC expression has been indicated by positive green fluorescence signals located in the cytoplasm, where it showed a reticular pattern suggesting a localization of SPC in association with the Golgi/ER complex [20]. These preliminary findings have been confirmed by the quantitative analysis of the surfactant proteins (SPC, B, A and D) performed by ELISA kit (Figure 2). These data indicated that the addition of secretome in HA solutions has improved the amount of expression obtained by the use of HA biomaterials alone. Lately, in previous work, our group demonstrated that HA in solution at low and medium MW can be used to stimulate the in vitro differentiation of MSCs into ATII cells secreting surfactant. Indeed, HA play in lung ECM the main role of water regulation in the interstitium, and it acts stabilizing the surfactant proteins on alveolar surface structure. Moreover, many specific biological functions of HA are related to the interaction of HA with its principal receptor CD44, expressed physiologically on MSCs surface. The CD44 receptor seems to be involved in pulmonary tissue remodeling. It has been reported, that HA directly triggers CD44 signal transduction via activation of merlin, PI3K and Erk signaling [15]. The secretome derived from Adipose mesenchymal stem cell has already shown increases osteoblastic metabolic activity and differentiation in vitro via activation of Smad/ERK/JNK (c-jun NH2-terminal kinase). When Secretome was added to in vitro cultures of osteoclasts also led to improved survival and differentiation [21,22]. However, no evidence has been shown so far of its regenerative potential in combination with HA. The main idea in the use of secretome in the regenerative medicine field regarding the consideration that secretome is esteemed safer than the MSC transplant. In fact, it involves a lower risk of emboli formation after intravenous infusion and lower risks of oncogenic or pathological transformation due to uncontrolled cell differentiation [23]. Furthermore, since the secretome reflects the cellular composition of the cell parents, it maintains the same immunoprivileged properties of MSCs, allowing the use of allogeneic preparations (including inter-species) without immune activation. Another important advantage to consider is that secretome treatment is not permanent and therefore, in the event of any adverse effects, it can be more easily stopped than cells [24]. Mechanisms of interaction between HA and the secretome are unknown to date and further investigation is required. However, the data collected here have demonstrated, for the first time, that their combination may lead to an improvement in the amount of lung surfactant protein C expression, along with the other surfactant proteins, compare the SAGM control. The cell viability has been demonstrated after 21 days. Indeed, the positive action of both HA and of the secretome separately in promoting and maintaining the cells viability is extensively know [25,26]. The synergistic effect of secretome and HA on wound healing properties can be observed since the HA + secre formulations presents a wound closure percentage higher compared to both the control with DMEM and with secretome alone and with the HA formulations without secretome. The role of HA in promoting wound healing, as a physiological constituent of human connective tissues, is already broadly recognized [27]. In fact, HA is able to improve cell migration speed and proliferation rate and is currently widely studied as a constituent in wound dressing applications [28]. Likewise, MSCs secretome has been applied in many pre-clinical studies as an acceptable alternative for therapies using replacement cells to treat wound healing [29]. It has been demonstrated that the HIF1α-associated redox signalling partner, when overexpressed in MSCs enhanced their paracrine therapeutic efficacy for the treatment of diabetic wounds. Indeed, another work has been indicated that the intracellular redox changes of human healthy and pathological-derived MSCs are important for the tissue regeneration, for example in the cardiac regeneration [30,31,32]. The results here collected confirm the positive effect of the combination of HA and secretome in wound healing activities of the solutions. In fact, all secretome-containing HA solutions tend to show a more efficient wound closure effect then the ones without secretome, with the only exception of HMWHA + secre formulation. The in vitro anti-inflammatory results revealed that the synergy between HA and secretome exerts preventive anti-inflammatory beneficial effects exhibiting regenerative properties. This behavior may be explained considering the beneficial effect of secretome and HA in the modulation of the inflammatory response [33,34].

## 4. Materials and Methods

### 4.1. Materials

Hyaluronic acid (HA) with a weight-average molecular weight (MW) of Low (L) 200, Medium (M) 500 and High (H) 1435 kDa were kindly provided by Altergon Italia s.r.l. Phosphate buffer saline (PBS) tablets without calcium and magnesium were obtained from MP Biomedicals Inc. hUCMSCs cells were extracted from the Wharton jelly of the umbilical cord kindly gifted by Ospedale Evangelico Betania (Naples, Italy), MRC-5 cells were purchased from ATCC. Dulbecco’s Modified Eagle′s—Medium (DMEM) (Microgem, Naples, Italy). SAGM (Lonza C-41 24) and Fetal Bovine Serum (FBS) were purchased from Lonza (Basel, Switzerland). Penicillin, streptomycin (10,000 U/ml) from Invitrogen and Life Technologies (Carlsbad, CA, USA) were employed. Trypsin and Ethylenediaminetetraacetic acid (EDTA) were from HiMedia (Mumbai, India). Formalin, bovine serum albumin (BSA) and 4′,6-diamidino-2-phenylindole (DAPI) were purchased from Sigma-Aldrich (St. Louis, MO, USA). SPC rabbit antibody from Abcam. FITC-conjugated anti-rabbit antibody (Millipore, Billerica, MA, USA). CD-73 antibody (Sigma Aldrich, St. Louis, MO, USA). Human pulmonary surfactant associated protein (SP) SP-A, SP-B, SPC and D ELISA kits were obtained from Elabscience.

### 4.2. HA/Secretome Solution Preparation

The HA concentration were obtained using L, M and HMW HA at 0.1%, 0.25%, 0.5% and 1% (*w*/*v*), respectively. For the cell viability experiment, HA was dissolved in DMEM and stirred for 3 h in order to guarantee the homogeneity of the solution. To carry out the differentiation experiments, a suitable amount of HA in the form of sodium salt was weighed and it was dissolved in a suitable volume of SAGM and then kept under stirring for 3 h. The solutions obtained from the previous procedure were sterilized by filtration using 0.22 micron filters. Secretome, (Lysosecretome) derived from human adipose MSCs, was purchased by Pharma Exceed S.r.l. and it has been used in all experimental conditions at the concentration of 0.2 mg/ml (2 × 10^4^ human adipose mesenchymal cells equivalent) solubilized in the HA (LMW, MMW and HMW) solutions implementing SAGM cell culture media.

### 4.3. Cell Culture

The hUCMSCs cells were grown in T-75 cell culture flask (Falcon, Italy) at early passages (1–6), in complete culture medium MEM, supplemented with 10% FBS and antibiotics (penicillin G sodium 100 U/mL, streptomycin 100 U/mL), in a humidified and controlled atmosphere at 37 °C and 5% CO^2^. The medium was changed every 3–4 days. When confluent growth was reached, the cells were detached with 0.25% trypsin—EDTA solution and washed twice with PBS. The resulting cell suspensions were centrifuged (5 min, 1000 rpm; BRK55/10 Centrifuge by Centurion Scientific Ltd., Stoughton, UK), the supernatant separated, and the cells re-suspended in fresh culture medium. Viable cells were counted using the TC20 automated Cell Counter (Biorad, Hercules, CA, USA).

### 4.4. Immunofluorescence

In order to assess the differentiation of hUCMSC cells, the qualitative expression of Surfactant Proteins C (SP−C) has been evaluated by immunofluorescence against SPC antibody. hUCMSCs were cultured for 21 days and seeded on fluorodish—35 mm (World Precision Instruments, Inc. Hitchin, UK), were fixed in 10% Formalin for 1 h, permeabilized with 0.1% Triton X-100, blocked with 1% BSA and incubated with SPC rabbit antibody (Abcam, Cambrige, UK) diluted in 1% BSA at 4 °C overnight. After washing with PBS three times, FITC-conjugated anti-rabbit antibody (Millipore, Billerica, MA, USA) was added to the cells for 3 h at room temperature. In parallel, qualitative expression of Cluster of Differentiation 73 (CD-73) has been evaluated by immunofluorescence against CD-73 antibody. hUCMSCs were cultured for 21 days and seeded on fluorodish—35 mm (World Precision Instruments, Inc.), were fixed in 10% Formalin for 1 h, permeabilized with 0.2% Tween 20 for 1 h, blocked with 1% BSA and incubated with CD-73 mouse antibody diluted in 1% PBS/BSA at 1:100 dilution for 2 h at 4 °C. Finally, for all samples, cell nuclei were stained with blue DAPI for 10 min at 37 °C. Samples were observed by confocal microscope system (Leica TCS SP5 MP) with a 63× oil immersion objective. Images acquired with a resolution of 1024 × 1024 pixels.

### 4.5. Surfactant Proteins Expression

To determine the quantitative expression of human pulmonary surfactant protein A-B-C and D the supernatants for analysis were collected after 21 days of exposure to the biomaterials, and then analyzed using SP A-B-C and D ELISA kits according to manufacturer protocol.

### 4.6. Cell Viability

In order to understand the optimum concentration of HA, cells viability was performed at different concentration of HA reported above. hUCMSC cells were seeded at a density of 3 × 10^3^ cells/ml on 96-wells (World Precision Instruments, Inc.). The cells were seeded for each well in triplicate cultured up to 24 h, then Alamar blue assay (AB) was performed by adding AB reagent to the samples (at 10% *v*/*v* with respect to the medium) and incubated at 37 °C for 4 h. The absorbance of the samples was measured using a spectrophotometer plate reader (Multilabel Counter, 1420 Victor, Perkin Elmer, Hopkinton, MA, USA) at 570 nm and 600 nm. AB is an indicator dye that incorporates an oxidation-reduction indicator that changes color in response to the chemical reduction in the growth medium, resulting from cell viability. Data are expressed as the percentage difference between treated and control to evaluate the percentage of reduction (Reduction %), which is calculated with the following formula:

$$Reduction\;\left(\%\right)=\frac{\left(O_{2}\times A_{1}\right)-\left(O_{1}\times A_{2}\right)}{\left(O_{2}\times P_{1}\right)-\left(O_{1}\times P_{2}\right)}\times 100$$

where *O*_1_ is the molar extinction coefficient (*E*) of oxidized AB at 570 nm; *O*_2_ is the *E* of oxidized AB at 600 nm; *A*_1_ is the absorbance of test wells at 570 nm; *A*_2_ is the absorbance of test wells at 600 nm; *P*_1_ is the absorbance of control well at 570 nm; and *P*_2_ is the absorbance of control well at 600 nm. The percentage of reduction for each sample was normalized to the percentage of reduction for the control to obtain the cell viability percentage [27].

### 4.7. Cell Migration Study

The wound healing potential of the biomaterials was assessed by wound healing assay by seeding hUCMSCs at a density of 1 × 10^4^ cells/ml on 96-wells (World Precision Instruments, Inc.) to obtain a monolayer of cells. Once adhered to the plate, wound healing assay was performed by scraping the cell monolayer in a straight line to create a “scratch’’ with a p10 pipet tip. Next, debris were removed by gently washing cells twice with 250 μL of sterile PBS 1X and then replaced with 200 μL of culture medium as control and with the solution of HA plus secretome and with HA and secretome alone. The sample were incubated in a humidified and controlled atmosphere at 37 °C and 5% CO_2_. In this assay, both control solutions and HA-containing ones were obtained using culture medium implemented with 2% FBS in order to minimize cell proliferation but to be sufficient in preventing apoptosis and/or cell detachment. To study hUCMSCs cells migration, images were acquired at time zero, immediately after the scratch, and after 3, 5 and 24 h for each sample in eightfold, using bright-field microscopy (‘Live Cell Imaging’ System, JuLI™ Stage). At the different times, wound width calculated as the average of 5 measurements in each sample between the edges of the scratch. Next, *rate of cells migration* (*R_M_*) was quantified by dividing the change in wound width (μm) by the time spent in migration (h):

$$R_{M}=\frac{W_{i}-W_{f}}{t}$$

where *W_i_* is initial wound width (μm) *W_f_* is final wound width (μm) and t is the duration of migration. Furthermore, wound area can be calculated by manually tracing in captured images the cell-free area, selecting an ad hoc ROI (*Region Of Interest*) [35]. The migration rate can be expressed as the change in the wound area as the percentage of area reduction or *wound closure*, as:

$$Wound\;closure\;\%=\left[\frac{A_{t=0h}-A_{t=\Delta h}}{A_{t=0h}}\right]\cdot 100\%$$

where *A_t_*_=0h_ is the area of the wound measured immediately after scratching (*t* = 0 h) and *A_t_*_=Δh_ is the area of the wound measured h hours after scratch is performed (i.e., 3 h, 5 h, 24 h) [36]. The percentage of closure clearly increases as the cells migrate over time to the scratch.

### 4.8. In Vitro Inflammatory Response

In order to assess the effect of HA and secretome on cell inflammatory response, basal levels and after-stress levels of inflammatory markers were quantified on experimental in vitro models of inflammation. Basal and post-inflammatory levels of interleukin 6 (IL-6) and interleukin-10 (IL-10) were measured. To test basal levels, hUCMSCs were seeded in tissue culture 96-well plates at a density of 1 × 10^5^ cells/well and incubating with DMEM, SAGM and Secretome as control and with LMWHA, MMWHA, HMWHA alone and plus Secretome for 3 days. Instead, to test post-stress inflammation hUCMSCs were previously seeded for two days in a 0.7% *v*/*v* DMEM solution of lipopolysaccharides (LPS). Cell supernatants were used to quantify IL amount through commercial ELISA kits (Elabscience) as reported on the manufacturer’s instructions [37].

### 4.9. Statistical Analysis

The results were expressed as mean ± standard deviation (SD). Data analysis was performed using Graphpad^®^ Prism 8 software. The repeated results were compared with the ordinary one-way analysis of variance (ANOVA) and a *p* value < 0.0001 was considered significant.

## 5. Conclusions

In this study, we successfully assessed the synergistic effect of HA and secretome able to support cellular viability and to induce pulmonary differentiations of hUCMSCs. In particular, the effect of different MW HA (L, M and H) has been investigated such as trophic factor, implementing the cell culture media. Our finding demonstrated that the combination of HA and secretome is able to promote MSCs viability and to increase the differentiation in ATII cells. Moreover, LMWHA + secre and MMWHA + secre solutions were particularly effective, compared the control samples or HMWHA solution, in enhancing the pulmonary regeneration as demonstrated by the strongly increment of the expression of the SPC along with the other surfactant proteins. Moreover, the synergistic effect of HA and secretome has been demonstrated affecting the cell migration activities and the capability to hinder the inflammatory reactions in basal conditions, exerting a preventive anti-inflammatory beneficial effect These outcomes suggested for the first time, that pulmonary tissue regeneration can be boosted by the presence of HA and secretome combined. This strategy could overcome the actual limits of stem cell-based therapies and improve the lung tissue repair.

## Data Availability

Not applicable.
